# Sustainable synthesis of multifunctional nanomaterials from rice wastes: a comprehensive review

**DOI:** 10.1007/s11356-023-29235-9

**Published:** 2023-08-14

**Authors:** Subhendu Chakroborty, Kaushik Pal, Nibedita Nath, Varun Singh, Arundhati Barik, Siba Soren, Pravati Panda, Nidhi Asthana, George Z. Kyzas

**Affiliations:** 1 Department of Basic Sciences, IITM, IES University, Madhya Pradesh, Bhopal, 462044 India; 2 Department of Physics, University Centre for Research and Development (UCRD), Chandigarh University, Gharuan, Mohali, Punjab 140413 India; 3 Department of Chemistry, D.S. Degree College, Laida, Sambalpur, Odisha India 768214; 4 Department of Chemistry, University Institute of Science (UIS), Chandigarh University, Mohali, Punjab 140413 India; 5 CIPET: Institute of Petrochemicals Technology [IPT], Bhubaneswar, Odisha India; 6 Department of Chemistry, Ravenshaw University, Cuttack, 753003 Odisha India; 7 Department of Basic Sciences, RIE, Bhubaneswar, India; 8 Graphic Era Deemed University, Dehradun, India; 9 Department of Chemistry, International Hellenic University, Kavala, Greece

**Keywords:** Waste management, Rice husk, Carbon, Cellulose, Lignin, Agriculture

## Abstract

More than 60% of India’s population relies on agriculture as their primary source of income, making it the nation’s most important economic sector. Rice husk (often abbreviated as RH) is one of the most typical by-products of agricultural production. Every five tonnes of rice that is harvested results in the production of one tonne of husk. The concept of recycling and reusing waste from agricultural production has received interest from a variety of environmental and industrial perspectives. A wide variety of nanomaterials, including nano-zeolite, nanocarbon, and nano-silica, have been discovered in agro-waste. From rice cultivation to the finished product, there was a by-product consisting of husk that comprised 20% of the overall weight, or RH. The percentage of silica in RH ash ranges from 60 to 40%, with the remaining percentage consisting of various minerals. As a direct consequence of this, several distinct approaches to generating and extracting nanomaterial from rice husk have been developed. Because it contains a significant amount of cellulose and lignin, RH is an excellent and economical source of carbon precursor. The goal of this chapter is to produce carbon-based nanomaterials from RH.

## Introduction

Nanotechnology is now a rising breakthrough with huge opportunities in a variety of fields, including electronics, healthcare, and the food sector. Nanofood, nanosensors, nano-packaging, nano-fertilizers, and nano-pesticides are some of the most recent nanotechnological innovations (Sahoo et al. 2021). Nowadays, numerous strategies have been developed for synthesizing various types of nanomaterials, but the Green synthesis method is one of the most effective since it uses naturally produced starting materials to provide a sustainable strategy for nanomaterial synthesis (Huston et al. 2021). Green techniques is a way of making nanomaterials that is clean, safe, cost-effective, and environmentally friendly. Nanomaterials are now made from various microorganisms, plants (Vanlalveni et al. 2021; Pal et al. 2022a, b), leaf extracts, and a variety of waste (Abdelbary and Abdelfattah 2020).

Nanomaterial has not only gained significance in the galaxy of research and development but has also caught up the eyes of industrial, environmental, and biomedical researchers worldwide (Chakroborty et al.^2023^; Nath et al. 2023a, b, c; Nath et al. 2023a, b, c; Pal et al. 2022a, b; Nath et al. 2023a, b, c). Carbon-based nanomaterials (CNMs) play a critical function among the various nanomaterials. In nanotechnology, carbon-based nanomaterials are particularly important (Nath et al. 2023a, b, c). Carbon nanostructures are divided into three types: three-dimensional graphite, two-dimensional graphene, one-dimensional carbon nanotubes, and zero-dimensional fullerenes (Villarreal et al. 2017; Jiang et al. 2017). The brightest stars among them are graphene, carbon nanotubes, and carbon nanofibers, which have the most promising nanotechnology applications (Siqueira and Oliveira 2017). CNMs have sparked a lot of interest in the research community because of their unusual features. Bio-imaging, tissue engineering, drug carriers, treatment of wastewater, catalysis, reduced pollution, biosensor, and energy storage system are some of the important applications of CNMs (Harrison and Atala 2007). The physical properties of synthetic nanomaterials are shown in Table 1.

**Table 1:** Physical properties of synthetic carbon nanomaterial

CNMs	Electrical conductivity (S cm^−1^)	Thermal conductivity (W m^−1^ K^−1^)	Tenacity	Hardness
Fullerene (0D)	10^−10^	0.4	Elastic	High
CNT (1D)	Structure dependent	35,00	Flexible, elastic	High
Graphene (2D)	~ 2000	4840–5300	Flexible, elastic	Uppermost (for single layer)
Graphite (3D)	Anisotropic 2–3 × 10^4^	Anisotropic 1500–2000, 5–10	Flexible, non-elastic	High

Agricultural waste is the term used to describe waste produced by agricultural activities (Zhang et al. 2012). The agro-wastes are created by a variety of agricultural activities and industries, including the handling of crop waste, pesticide, insecticide, and herbicide waste, as well as the disposal of animal excreta and carcasses (Zhang et al. 2012; Ali et al. 2017b, a). However, there is a range of organic and mineral resources in agricultural wastes which is recovered and reused for a number of purposes, such as starting material for energy production and remediation of harmful pollutants (Obi et al. 2016). Due to growing agricultural productivity in recent years, agricultural wastes have expanded along with the global population. A considerable number of environmental issues are brought on by the enormous amount of agricultural waste (*998 million tonnes) (Agamuthu 2009).

Furthermore, a farm produces 5.27 kg of organic waste per 1000 kg of living weight, which accounts for 80% of all solid waste (Hadiya et al. 2018). Significant policymaker concerns for sustainable development and green agriculture have recently been muzzled by Agricultural Waste Management (AWM) (Hai and Tuyet 2010, Hegde et al. 2015, Ali et al. 2014b, a). Numerous procedures and factors were looked at in terms of managing waste management for prospective reuse and lowering the threat of resource pollution. The use of technology and inducements and a change in perspective and attitudes are only a few of the additional efforts needed to control AWM. There are numerous potential uses for AWM, including the application of fertilizer, anaerobic digestion, animal feed, adsorbent materials, source of heat, direct burning, and the production of nanomaterial (Obi et al. 2016).

Rice is one of the world’s oldest crops grown for centuries. Recently, it is grown in over 100 countries, and almost half the world’s population eats it as a basic diet. Population growth and economic expansion in developing nations are expected to boost global rice consumption from 480 million tonnes (Mt) of milling rice in 2014 to roughly 550 Mt by 2030 (Rice Research Institute 2016). A huge amount of trash is produced during the production of rice-based foods. This garbage has a disposal difficulty, which has negative consequences for the environment and human health. Rice trash contains husk, straw, bran, ash, and broken rice (Moraes et al. 2014). RH is a common rice waste product, that is, used to create nanomaterials employing green technology. This chapter focussed on the synthesis of carbon-based nanomaterials using RH.

## Rice wastes

### Rice husk as waste

RH is the outer coat of rice seeds that are cylindrical in shape, and its size ranges between 4 to 10 mm depending on the seed variety employed. RH is made up of 41.92% carbon, 6.34% hydrogen, 1.85% nitrogen, and 0.47% sulfur (Biswas et al. 2017). Rice husk is a global product of about 1 million tonnes per year (Liou and Yang 2011). Rice husk is mostly composed of hydrated silicon and organic compounds such as cellulose (55–60 wt%), including lignin and hemicelluloses and cellulose (22 wt%). The white ash resulting from the moderate-temperature burning of this feedstock comprises 87–97% amorphous silica and a small number of metallic contaminants (An et al. 2010). Humans should not consume this product. Environmental concerns are created by dumping the ash and partly bored husk (An et al. 2010; Jung et al. 2008, Chaudhary and Jollands 2004). When 1 tonne of rice husk is burned in the field, 0.15 kg of CO_2_ is released into the environment, whereas rice husks yield 90 g of methane gas when they decompose naturally in the soil (Umeda and Kondoh 2010). Many rice-producing nations burn rice husks in outdoor dumps, which can pollute the air. Some discard them in open landfills, where the rice husks degrade and eventually produce methane, a greenhouse gas contributing to global warming (Omatola and Onojah 2009). Because of the growing amount of waste, a number of research groups are looking for ways to convert it into useful products.

It was discovered that silica was substantially dispersed in the husk’s outer surface using back-scattered electrons and X-ray imaging analysis of the RH, with less presence in the mid-region and inner epidermis (Stroeven et al. 1999). RH exhibits a characteristic globular, well-organized, corrugated outer surface, as a scanning electron microscopy (SEM) study determined. SEM imaging of the side section of RH revealed an interlayer between the inner and outer surfaces. The interlayer features multiple pores with a diameter of 10 µm, is loose and honeycombed, and is constructed of interlaced plates and sheets (Jiang 2010). RH usually is 8 to 10 mm long, 2 to 3 mm wide, and 0.2 mm thick (Fang et al. 2004). Actual densities range from 670 to 740 kg/m^3^, while RH has a bulk density of between 100 and 160 kg/m^3^. However, RH can only be compressed to 400 kg/m^3^. Approximately 80% of RH’s composition is organic, and 20% is inorganic. Crude protein and fat are deficient, ranging from 2.0 to 2.8% and 0.3 to 0.8%, respectively, compared to crude fiber. It consists mostly of lignin, which ranges from 20.4 to 33.7%, hemicellulose, which ranges from 14.0 to 28.6%, and hemicellulose, which ranges from 28.6 to 41.5% (Champagne et al. 2004; Quispe et al. 2017).

RH is an excellent option for a starting material because it is a biomass, renewable material, is very inexpensive, and can be produced in massive amounts. According to a recent literature review, some research groups have synthesized silica and carbon-based nanomaterials from RH for environmental and economic reasons (Seyfferth et al. 2016; Vargas et al. 2019). As per Jonathan et al., open and closed burning of rice husk-derived silica indicated distinct phases of crystalline and amorphous silica (SiO_2_) accordingly (Liu et al. 2013; Patil et al. 2014). RH is an essential feedstock for producing CNMs such as biochar, graphene, and graphene oxide, carbon nanotubes (CNTs) (Wang et al. 2018a, b; Guan et al. 2019; Asnawi et al. 2018). Wang et al. prepared graphene quantum dots from RH for Fe^3+^ sensing (Wang et al. 2018a, b).

### Rice husk ash

After being burned, RH produces rice husk ash (RHA), which weighs 17 to 20% less than the lightweight and clumsy RH. The RHA is a porous substance with a roughly 180–200 kg m^−3^ density. Depending on the circumstances of the combustion process, RHA is divided into two categories: black RHA and white RHA (Ugheoke & Mamat 2012). White RHA is produced by carefully controlling the pyrolysis of RH in the air. White RHA typically consists of hydrated, amorphous, pure SiO_2_ (> 95%) with a high porosity, and reactive surface (Vlaev et al. 2003). In the meantime, the production of black RHA can be achieved by carefully burning RH in an inert environment such as nitrogen (Ghaly & Mansaray 1999). 1.22% K_2_O, 1.28% Fe_2_O_3_, 18.24%C, 1.20% Al_2_O_3_, 89% SiO_2_, and 1% CaO are the chemical composition of RHA (Mohamed et al. 2015). Fe, Ca, Cu, K, Mg, Mn, Na, and Zn traces are also observed (Zou and Yang 2019).

**Fig. 1:**
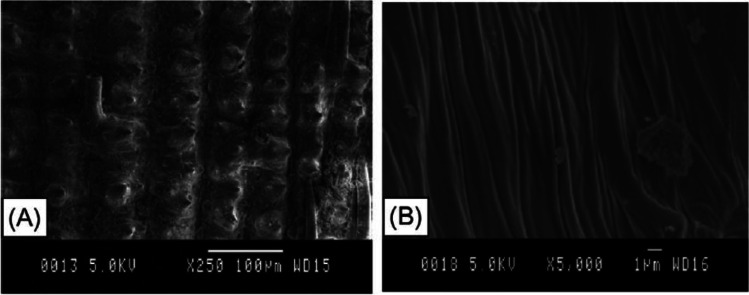
Shows a SEM image of the (**A**) external surface morphology, and (**B**) the interior surface (Xu et al. 2012)

**Fig. 2:**
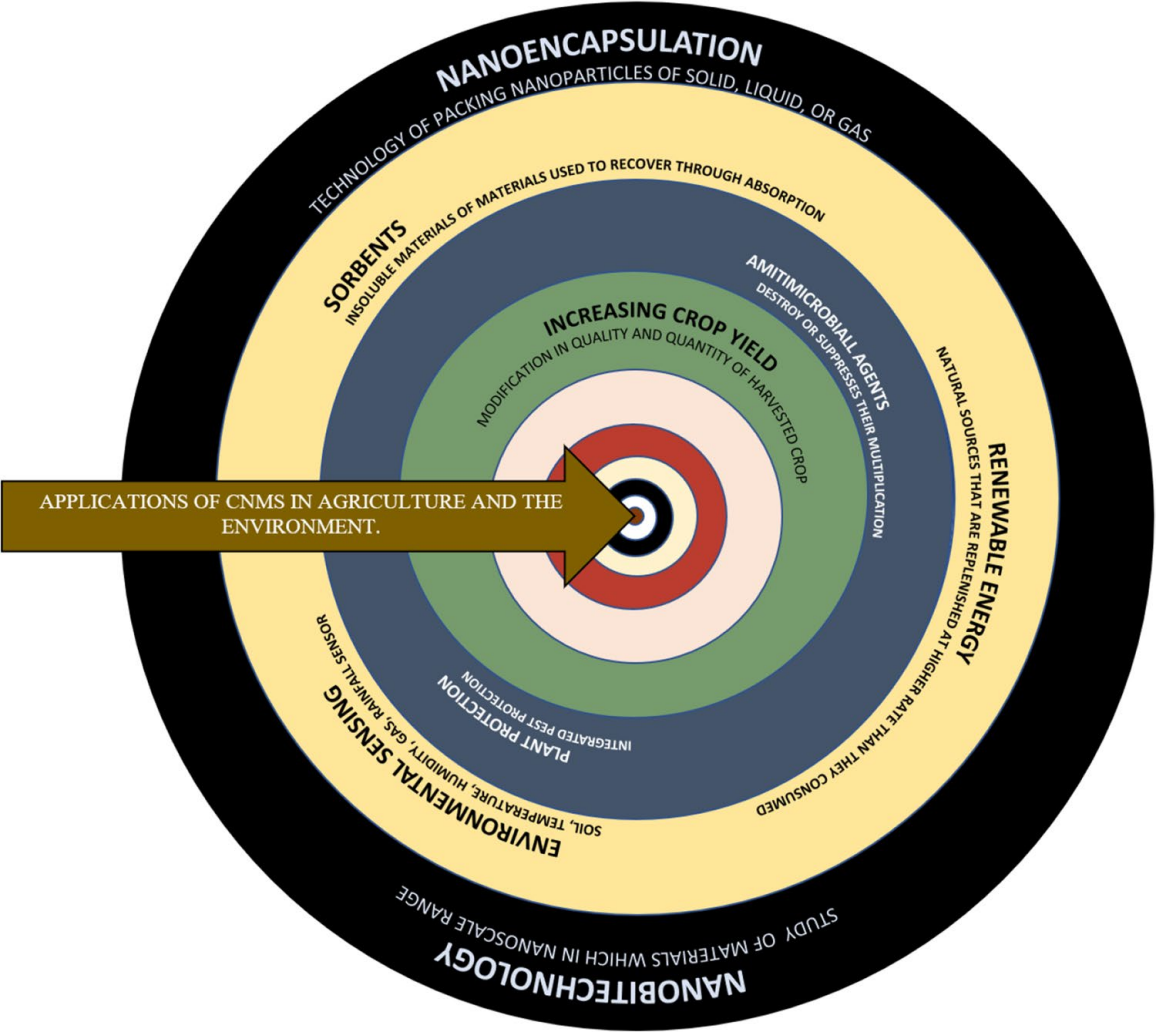
Applications of CNMs in agriculture and the environment

While scanning electron microscope (SEM) image of RHA (Fig. 2) produced by the combustion of RH at 600 °C for 2 h in an electric oven, major components of RHA survived air combustion unaffected, but minor components sustained structural damage (Fig. 1A). Because both sides of RHA display a dense structure, it is indeed possible that a compacted membrane with no micropores covers both the external and interior surfaces (Fig. 1A, B) (Xu et al. 2012).

Ouyang and Chen discovered the three-layer concept while analyzing the microstructure of RHA utilizing SEM and transmission electron microscopy (TEM) studies. The interlacing of the fiber sheet creates micropores in RHA, which are reliant on the RH structure but independent of the combustion conditions, and nanopores, which are generated by nano-SiO_2_ particles and are around 50 nm in size and depending on the combustion temperatures (Ouyang and Chen 2003). SiO_2_ particles and nanopores at the nanoscale are the primary contributors to RHA’s high surface area and activities. Along with Kim, in the diffractogram, there is a noticeable smooth bump between the angles of 15° and 35°, proving that pyrolysis changed the crystalline structure of cellulose into an amorphous, chaotic, disordered structure. Kim and other research groups reported that the crystalline structure of cellulose was altered by pyrolysis into an amorphous, chaotic, disorganized form (Kim et al. 2008).

## Nanomaterial synthesis from rice husk

Due to their numerous fascinating physical and chemical characteristics, such as high quantum yield, high surface area, and inventive morphological structure, nanomaterials are widely created and used for a number of applications (Naddaf et al. 2019; Emran et al. 2019, 2017; Emran et al. 2018a, b, c, d, e, f, g; Emran et al. 2018a, b, c, d, e, f, g; Emran et al. 2018a, b, c, d, e, f, g; Gomaa et al. 2017; Thalji et al. 2019; Ali et al. 2018, 2013; Fouad et al. 2011). They are strong contenders for a variety of applications in numerous fields of science and nanotechnology due to these intriguing characteristics (Emran et al. 2018a, b, c, d, e, f, g; Emran et al. 2018a, b, c, d, e, f, g; Emran et al. 2018a, b, c, d, e, f, g; Emran et al. 2018a, b, c, d, e, f, g; Gomaa et al. 2018; Barhoum et al. 2019; Ali et al. 2017b, a, 2014b, Abdel Ghafar et al. 2015). Removing nanomaterials from natural bio-resources, such as bacteria, plants, waste, and agricultural residues, has recently garnered much attention (Griffin et al. 2018). This strategy differs from traditional strategies in a number of ways, including flexibility, economy, a higher level of safety, and less negative environmental impact. In particular, producing nanomaterials from agricultural waste is viewed as a cost-effective alternative to industrial raw materials and a renewable source for mitigating environmental decay issues. Nanomaterials made from agricultural waste include nanosilica and nanocarbon (Mor et al. 2017).

Due to its high silica concentration, RH is currently a source for many silicon compounds, including silicon nitride, silicon tetrachloride, silicon carbide, silica, zeolite, and pure silicon. One of the many materials that are readily accessible and used in a variety of applications, including thixotropic agents, thermal insulators, and composite filler, is silica (Pek et al. 2008). The design of biosensors, drug delivery, cell labeling, imaging, and separation have all benefited from the use of silica nanoparticles (Si NPs) (Fouad et al. 2011, Ali et al. 2016, Fouad et al. 2012, Tang and Cheng 2013, Singh et al. 2017a, b, Tao 2014, Ali et al. 2020). The synthesis of Si NPs made considerable use of RH/RHA as well as other agricultural by-products and wastes (Naddaf et al. 2019; Mor et al. 2017; Pouroutzidou et al. 2019).

RH was used as a precursor to silica to make mesoporous silica NPs for drug delivery (Purwaningsih et al. 2019). SiO_2_NPs that are black were also created using RH (Almeida et al. 2019). Matsumoto et al. produced brilliant Si NPs and investigated their optical properties using Mg reduction of SiO_2_NPs extracted from RH (Matsumoto et al. 2018).

Rice husk is a valuable source of cellulose nanofiber (CNF). The CNFs produced from rice husks showed strong fluorescence emission potential with outstanding quantum yield in contrast to their thermal properties and crystalline nature (Moon et al. 2011; Leung et al. 2013). Blue fluorescence was visible when the held CNF was exposed to UV light. The mechanical treatment and acid digestion of rice husk led to highly fluorescent CNFs in two rice varieties, Ahu and Boro (*Oryza sativa* L. ssp. *indica*). These CNFs have syringyl and phenyl coumarone groups, these are what give them their fluorescence capabilities and a little amount of lignin (Kalita et al. 2015). Porous carbon nano-onions (CNO), activated using renewable RH as a carbon precursor, are made using straightforward nickel-assisted graphitization (Jin et al. 2019). On silica made from rice husks, AgNPs with a diameter of 25 nm and a surface area of 514 m^2^ g^−1^ were synthesized and studied (Andas and Adam 2016). A different study isolated silica and magnesium oxide from RH and synthesized into 80–85 nm forsterite (Mg_2_ SiO_4_) NPs using a solid-state technique (Mathur et al. 2018). Bathla et al. produced silica nanowires with a diameter of 15 to 35 nm and a length of about 0.5 µm using rice husk ash (Bathla et al. 2018). Rice husk, for example, can be used in a microwave-assisted technique by Praneetha and Murugan to form SiO_2_ (Praneetha and Murugan 2015).

There is a lot of interest in CNMs for various potential uses, including power storage, sensors, catalyst, and water treatment. The applications for the CNMs in agronomic practices and also in the environmental application are shown in Fig. 2. There are numerous types of CNMs, including nanotubes, nanospheres, fullerene, and graphene (Emran et al. 2018a, b, c, d, e, f, g; Emran et al. 2018a, b, c, d, e, f, g; Emran et al. 2018a, b, c, d, e, f, g; Emran et al. 2018a, b, c, d, e, f, g). We demonstrate the creation of carbon nanoparticles from RH in this chapter.

## Synthesis of carbon-based nanomaterial from rice husk

A family of synthetic nanomaterials called agro waste-derived CNMs has numerous uses because of its outstanding physical and chemical properties (Mukherjee et al. 2016).

Carbon-based materials have excellent physiochemical properties that are used for a wide range of applications (Greil 2015; Zhang et al. 2013). Carbon-based materials can often be categorized into four types based on their morphological structures: fullerenes and carbon dots are zero-dimensional (0D) materials. Carbon nanotubes (CNTs) are examples of one-dimensional (1D) materials. Graphene and graphite are two-dimensional (2D) materials (Villarreal et al. 2017; Jiang et al. 2017). CNTs are a material made of carbon with remarkable qualities like great chemical and mechanical stability and interesting electrical conductivity. In addition, the 2D material graphene has remarkable features, including graphene edges and highly transmitted light (Wang et al. 2015a, b). The production and application of CNMs, such as CNTs and graphene, for items like electronic devices, sensors, composite materials, and energy storage devices have both received a great deal of attention up to this point (Emran et al. 2017; Emran et al. 2018a, b, c, d, e, f, g; Emran et al. 2018a, b, c, d, e, f, g; Akhtar et al. 2017). The environmentally friendly synthesis of carbon-based materials utilizing waste materials has received much attention. RH is a good and plentiful source of carbon nanomaterials due to its high cellulose and lignin content (Kure et al. 2017). Table 2 lists the numerous carbon nanomaterials made from different biowaste and their synthesis methods.

**Table 2:** Summary of CNM made from inexpensive biowaste

Carbon generated	Bio waste	Synthesis method	Reference
Thin graphene (2–6 layers) MWCNTs	Rice husk	Microwave CVD	(Wang et al. 2015a, b)
CNTs	Rice straw	Conventional CVD	(Fathy 2017)
CNTs	Wood sawdust	Pyrolysis	(Bernd et al. 2017)
Graphene sheets	Rice husk	Combustion/chemical activation with KOH	(Muramatsu et al. 2014; Rhee et al. 2015, 2017)
C-dots	Rice husk	Pyrolysis	(Wang et al. 2016)
GQDs	Rice husk	Hydrothermal, pyrolysis	(Wang et al. 2016)
Graphene	Rice husks	Carbonization	(Sankar et al. 2017)
Graphene	Wheat straw	Hydrothermal and graphitization	(Chen et al. 2016)
Graphene	Peanut shells	Mechanical exfoliation	(Purkait et al. 2017)
GQDs	Rice husk	Carbonization and refluxed	(Mahmoud et al. 2020)
Graphene oxide	Coconut shell waste	Modified Hummers method	(Sujiono et al. 2020)
MWCNTs	Bamboo charcoals	CVD	(Zhu et al. 2012)

### Synthesis of CNTs

Since their discovery by Iijima in 1991, carbon nanotubes (CNTs) have been one of the most renowned one-dimensional carbon nanomaterials (Iijima 1991). Due to their one-dimensional structure and exceptional capabilities as electrical, chemical, thermal, mechanical, optical, and electronic, CNT materials have attracted a lot of attention from researchers (de Volder et al. 2013, Chu et al. 2010, Anantram & Leonard 2006, Prasek et al. 2011, Qi et al. 2003, Zhang and Li 2009). As a result of these properties, CNTs are suitable for a variety of applications, including electron field emission, energy storage and production, hydrogen storage, nanocomposites, separation, catalyst support, and drug delivery. CNTs can be made in single-walled carbon nanotubes (SWCNTs) and multiwalled carbon nanotubes (MWCNTs), each with a few to several nanometers in diameter. The CVD method is often considered the most suitable commercial production method. The choice of carbon-containing feedstocks, catalyst, substrate, and the required energy consumption during the CVD process all play a role in CNT synthesis (Birὀ et al 2001). As CNT precursors, various petroleum hydrocarbons in gaseous forms, such as methane, ethylene, and acetylene, as well as liquid conditions, such as alcohols, benzene, xylene, and cyclohexane, have been widely employed (Birὀ et al 2001, Baughman et al. 2002, Jha et al. 2013, Dai 2002, Serp, & Figueiredo 2009, Yu et al. 2010, Qi et al. 2003).

**Fig. 3:**
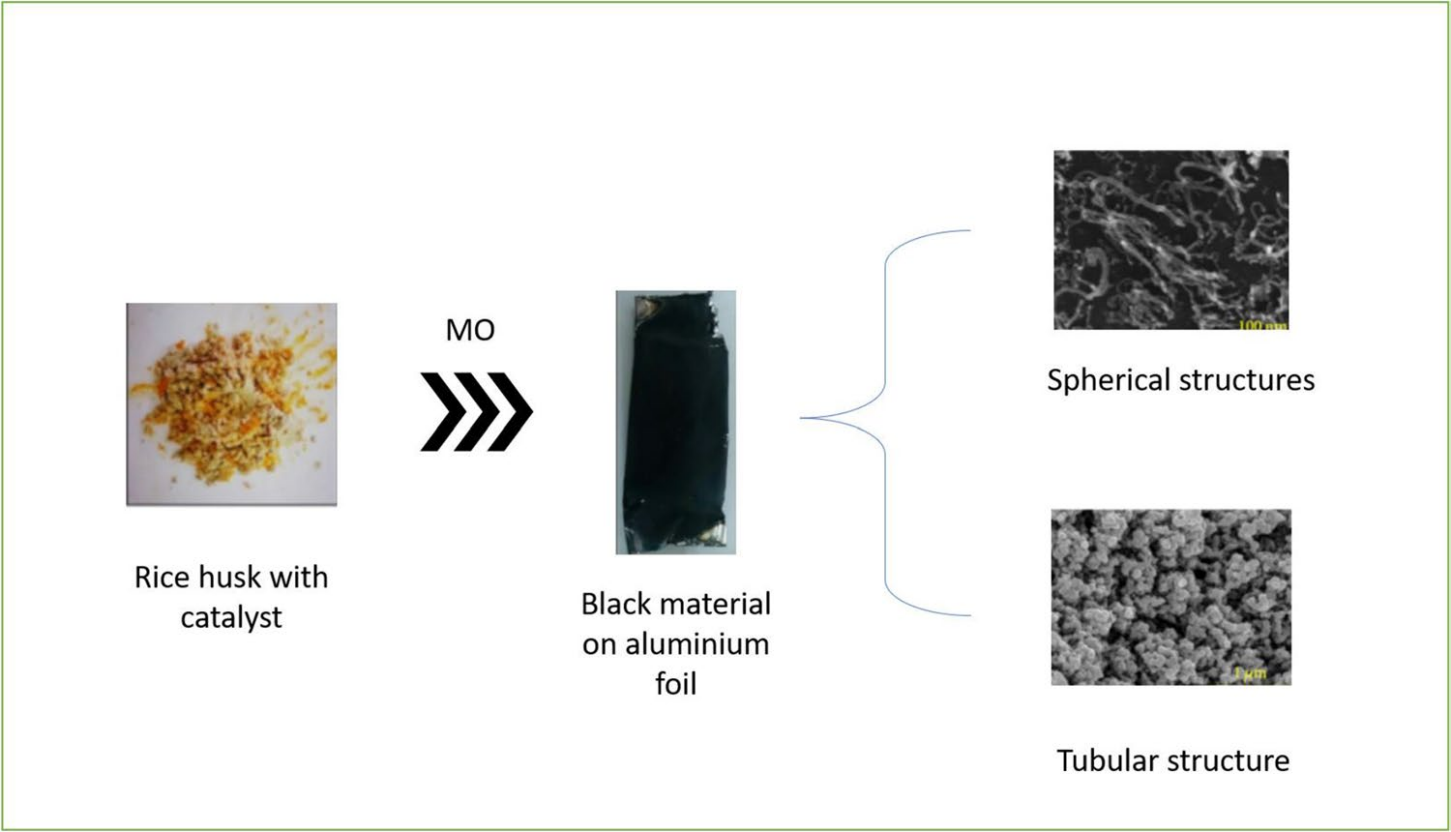
Conversion of RH into spherical and tubular structures of carbon CNTs (Wang et al. 2015a, b)

Much research has been done to promote green technology and increase CNT mass manufacturing in response to growing environmental concerns and the CNT market. Few studies have recently concentrated on the improvement and simplicity of CNT synthesis using agro-waste. CNTs were synthesized from waste RH powders using the microwave (MW) method. A carbon source, catalyst, and microwave oven are required to induce plasma. In the presence of ferrocene, the plasma improves and accelerates the catalytic degradation of RH (Fig. 3) (Asnawi et al. 2018). MW plasma irradiation (MWPI) method was introduced by Wang et al. for synthesizing graphene-CNTs (g-CNTs) from RH under H_2_ and Ar flow (Wang et al. 2015a, b).

Zhang’s research group employed a polyacrylonitrile (PAN)-aided electrospray approach to synthesize Si/nitrogen-doped C/CNT (SNCC) nano/micro-structured spheres for the first time, employing RH-derived Si NPs as a raw material. RH-derived Si NPs with diameters of 50 nm were uniformly dispersed and incorporated in an N-doped carbon matrix that was interwoven and connected by a CNT cross-linking network, resulting in microspheres having diameters of 3.2 ± 0.8 μm (Zhang et al. 2016).

For the synthesis of N-doping of CNTs, polypyrrole (PPy) was used as the carbon precursor, while mixed salts composed of sodium chloride and zinc chloride were used as the activating agent (NCNs-A). It was determined how the physicochemical and electrochemical characteristics of NCNs-A were affected by the individual salts ZnCl_2_ and NaCl, as well as the carbonization temperature and the mass ratio of PPy to the combined salts. The resultant NCNs-A exhibits good capacitive performance, thanks to their high SSA, broad pore architecture, hollow tubular shape, and high quantity of N-doping when used as electrode material for supercapacitors. Additionally, this NCNs-A has a broad pore architecture (Zong et al. 2020).

### Synthesis of graphene

Graphene seems to have become a material of interest and has piqued the scientific interest of several research communities around the globe since its discovery in 2004 by Andre Geim and Novoselov at the University of Manchester (Novoselov et al. 2004). Graphene is a 2D carbon sheet with a hexagon lattice structure that is a single atom thick. The sp2 hybridized carbon forms a structure that resembles a compact honeycomb and functions as a framework for a large number of nanocarbon. Stacking results in the formation of three-dimensional graphite, whereas rolling or coiling results in one-dimensional nanotubes and zero-dimensional fullerenes (Allen et al. 2010). The chemical inertness of the material, the quantum Hall effect, high carrier mobility, ambipolar field effect, and super hydrophobicity are only a few of the properties that have brought the material to the forefront of attention (Choi et al. 2010). These peculiar qualities led to the discovery of a great deal of intriguing physics and led researchers to hypothesize that graphene may be utilized in various cutting-edge electronic applications.

Functionalized graphene and non-functionalized graphene are two different forms of graphene, in contrast to non-functionalized graphene, single-layer graphene, and graphene sheets, functionalized graphene oxide (GO), and reduced graphene oxide (Homaeigohar & Elbahri 2017).

**Fig. 4:**
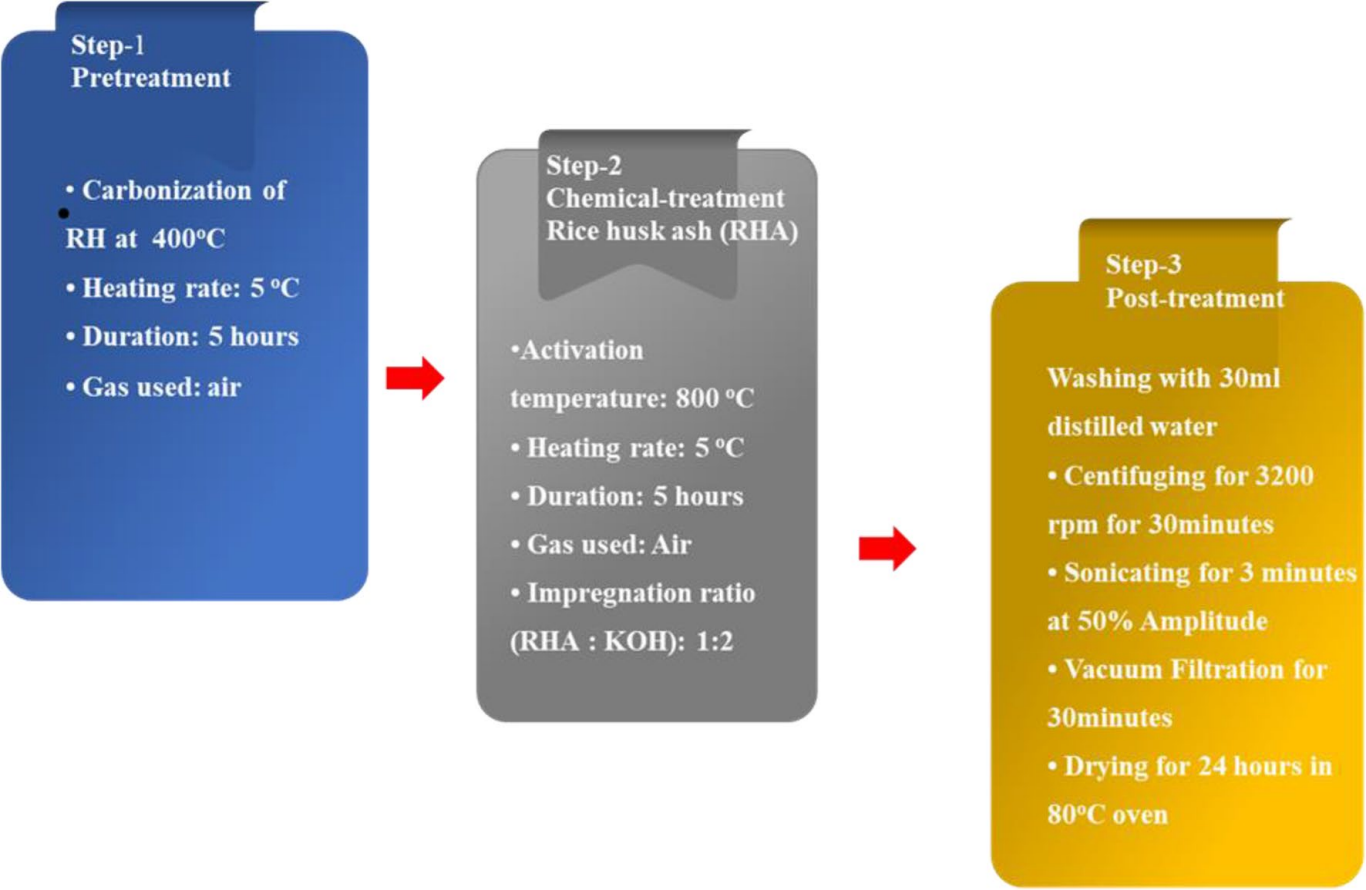
Experimental flowchart of synthesis graphene derived from rice husk

Through the activation of RHA with KOH, Muramatsu et al. showed a simple, cost-effective, and scalable approach for synthesizing graphene with stable and atomically clean edges (Wang et al. 2016). Graphene was successfully produced using this approach by activating (RHA) using KOH at 800 °C with a 1:2 impregnation ratio, as shown in (Fig. 4) (Ismail et al. 2019). RHA-derived graphene was also synthesized by the Othman research group utilizing KOH as a dehydrating agent at 800 °C and a (1:5) impregnation ratio for CH_4_ adsorption, which will be employed for natural gas storage or transit in the coming years (Che Othman et al. 2020).

Singh et al. developed a more cost-effective synthesis technique. They used RHA as a carbon source for graphene production and KOH as an activating agent in our procedure. The acquired results revealed that few-layer graphene was successfully synthesized. To test electrochemical properties for energy storage applications, cyclic voltammetry was used. RH was burned into the air to make RHA, which was then.

Moreover, 3 mg of RHA was combined with 15 gm of KOH and ground for 15 min. A mixture of rice husk and KOH was compacted in a porcelain crucible. This crucible was then wrapped in ceramic wool and placed inside a bigger graphite crucible. A sufficient amount of sacrificial RHA was applied to the top of the graphite crucible to establish a barrier preventing sample oxidation inside the porcelain crucible. The sample was annealing at 900 °C in a muffle furnace for 2 h. Following this activation process, the sample was rinsed with deionized water to eliminate excess KOH before being dried for 24 h at 100 °C (Singh et al.2017a, b). Sankar and his co-worker prepared Brown graphene nanosheets and explored their electrochemical energy-storage ability from the perspective of their applicability as an electrode material. Despite its simplicity, the production approach yields crystalline ultrathin graphene nanosheets with a low defect density (Mahmoud et al. 2020).

RH is used to synthesize graphene using a simple microwave technique. RH is first rinsed with distilled water by sonication for 1 h. The RH was then dried for 24 h in the open air at 40 °C. The mechanical approach is used to turn the dried RH into powder. The catalyst is ferrocene Fe(C_5_H_5_)_2_, and the ferrocene and ethanol are combined at three different levels with a magnetic stirrer (Kumar et al. 2020). Graphene layers were produced by activating the rice husk with potassium hydroxide and then desilicating it in an alkaline solution. Rice husk samples were carbonized under the following circumstances: rice husk/KOH weight-to-weight ratio was 1/4, and activation duration was 2 h at 850 °C. The NaOH desilication solution had a 1 M concentration. Raman spectroscopy was used to analyze the collected samples; the peaks indicate the presence of graphene layers. The product yielded less than 3% of its weight (Azizovna et al. 2017).

Rice husk is the feedstock used in the carbonization and KOH activation procedure to produce graphene. After that, graphene is made and investigated. It is completely even on the surface and has functional groups along its edges. In both the presence and absence of a compatibilizer, graphene is melt-mixed with polypropylene (PP) and polyamine-6 (PA6) in a ratio of 50/50 (wt/wt). The maleic anhydride (MA) grafted onto the polypropylene serves as the compatibilizer (PP-g-MA). The findings of the FTIR spectroscopy indicate that the PA6 phase and PP-g-MA react with one another. Localization of graphene occurs in the PA6 phase of a PP/PA6 mix with a weight-to-weight ratio of 50/50 (wt/wt) and its nanocomposites in both the presence and absence of PP-g-MA (Tanniru and Tambe 2022).

Rhee et al. use graphene made from rice husk to study the electrical characteristics of cement mortar (GRHs). It looks at improved varieties of agricultural waste-derived rice husk-derived graphene-like materials. The control specimens in this experiment were chosen based on the size and shape of their nano component to analyze the performance of GRH within cement mortar. This set of materials included multiwalled carbon nanotubes (MWCNTs), MWCNTs decorated with COOH, and carbon nanofibers with significantly higher aspect ratios because of their 1D structures. The 2D planar or corrugated-planar xGnP M15, xGnP C650, and GRH materials made comprised the rest of the comparison group. Electrical conductivity tests have shown that 1D structured inclusions outperform 2D structured inclusions. According to measurements of the change in volume resistivity vs. stress and strain, the electrical performance of the GRH composite fell within a moderate range comparable to that of carbon fiber (Rhee et al. 2015).

Sekar and his team recently used the KOH activation method to create corrugated graphene nanosheets from rice husk biomass. The RH-CG nanosheets displayed intense electric conductivity and a huge surface area after they were heated to 700 °C. The outstanding HER activities with a small overpotential (9 mV at 10 mA/cm^2^) and a small Tafel slope (31 mV/dec) were attained when the RH-CG nanosheets were used as a HER electrocatalyst in 0.5 M H_2_SO_4_. The findings provide a novel method for realizing an excellent electrocatalyst made of biomass for highly effective hydrogen production (Sekar et al. 2022).

RH was used as the primary raw material in a modified version of the Hummers’ method that was used to produce GO. The homogeneity of the synthesis process was evaluated using ground pencil leads as a control powder of the initial raw material to ensure that the powder was representative of the material as a whole. The pluronic F127 solution acted as the pore template during the precipitated technique used to create the TiO_2_ microspheres. Microspheres of TiO_2_ were mixed with GO derived from RH (GO-RH) to develop composites with weight-to-volume ratios of 3:1, 2:2, and 1:3. According to the results of the characterization, GO-RH produced a ternary phase material that contained graphite oxide, silica, and GO (Manpetch et al. 2022).

### Synthesis of carbon dots

Carbon dots (CDs) are one special type of carbon nanoparticle discovered in 2004 when extracting single-walled carbon nanotubes. These nanomaterials are fewer than 10 nm in size and feature a number of unique properties, such as strong biocompatibility, low toxicity, high water solubility, and a peculiar fluorescence property (Sharon & Mewada 2018). CDs are similar to graphene nanomaterials in that they have an amorphous structure (Fadllan et al. 2017). CDs are dispersive quasi-spherical nanoparticles made of carbon cores and shells of functional groups like the carboxyl group and the amino group and are a shining star of zero-dimensional carbon materials (Jing et al. 2019; Lim et al. 2015; Baker & Baker. 2010). The primary types of CDs are graphene quantum dots (GQDs) and carbon quantum dots (CQDs), which show significant potential in a variety of applications such as photocatalytic activity, biomedical imaging, sensing, drug delivery, and energy storage (Zhu et al. 2020). The sp^2^ and sp^3^ hybridized carbon atoms in these nanomaterials enable further exploration of their adjustable characteristics (Kokorina et al. 2017). CQDs can be synthesized in various ways, involving top-down and bottom-up strategies.

The CDs were produced by sonicating 10 gm of prepared dry husk in a solution of 2.5 M 50 mL HNO_3_ for 15 min. After placing the mixture in an autoclave lined with Teflon, it was heated to a temperature of 200 °C for 6 h. After waiting for the dark brown liquid to reach room temperature, a solution of 10 M NaOH was used to neutralize it, and then, it was filtered. The residue was set aside and later utilized in the production of mesoporous silica. The filtrate was centrifuged for 15 min at a speed of 10,000 rpm in order to remove any large particles. After using a dialysis membrane to filter the fluid, it was then freeze-dried to produce brown solid carbon dots weighing 0.93 gm and having a concentration of 9.3%. According to the findings of DFT calculations, UV–vis testing, and electronic nose analysis, the created carbon dots are suitable for real-time sensing of alcohols and VOCs (Thongsai et al. 2019).

**Fig. 5:**
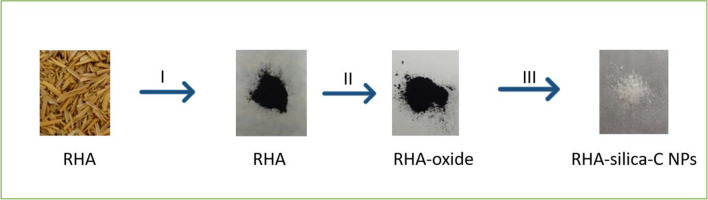
RHs to RHA and RH-silica-C NPs transformation I: pyrolysis under nitrogen atmosphere; II: oxidation; III: carbon grafting, framework cutting, and oxygen-containing group reduction (Wang et al. 2017)

CQDs were produced from RH using a hydrothermal process that required varying proportions of amine and carboxyl group sources in ethylenediamine and ascorbic acid, respectively. The functionalized CQDs were evaluated by HRTEM, FTIR, UV–vis, photoluminescence PL, and XPS. To remove cadmium from an aqueous solution, functionalized CQDs with the right amount of EDA and ascorbic acid will be utilized (Abidin et al. 2020a, b). Wang et al. used RH as a precursor to synthesize high-yield CQD-grafted silica NPs (Si–C NPs). The pyrolysis of water-rinsed rice husks (5 g) inside a tubular furnace in nitrogen atmosphere at 700 °C for 2 h results in the production of RHA among both silica and carbon. To remove silica, RHA was reacted using 1.0 M NaOH at 100 °C for 2 h. Then, in two steps, RHA with solely carbon content was oxidized with H_2_SO_4_ and HNO_3_; every step was performed by sonication, producing inside a black dispersion. It is proceeded by suction filtering using a 0.22 mm microporous membrane, which is subsequently rinsed with deionized (DI) water numerous times. Before being put into a 40-mL Teflon-lined autoclave, the black substance was mixed with 30-mL DI water. The dispersion undergoes a 10-h hydrothermal treatment at 200 °C. Then, it is cooled to room temperature and filtered once more. The filtrate successfully recognized RH-generated CQDs. A solid CQDs powder is created by evaporating the filtrate at 40 °C in a vacuum oven (Fig. 5) (Wang et al. 2017).

The prior method was modified by Wongso et al. to produce CQDs from RH. Without using any functionalizing agents, CQDs were synthesized, and this process was modified by adding NaOH to adjust the pH from 0 to 14. In this mechanism, intercalation was followed by exfoliation. Intercalation begins after RHA is diffused in a solution containing H_2_SO_4_ and HNO_3_. Following that, exfoliation begins by rinsing RHA in deionized water, resulting in CQDs. HR-TEM and EDX techniques were used to investigate the material. The size of CQDs and the quantity of oxygen-containing groups throughout the surface are affected by pH changes, while the sample’s crystal structure is unaffected. Due to the presence of NaOH, which interacts with HNO_3_ to produce NaNO_3_, different sizes appear. The excess NaOH serves as an oxidant and speeds up the intercalation process of RHA when HNO_3_ is entirely treated with it (at pH neutral). Changing the pH of the synthesis helps to broaden the emission spectrum of CQDs from green to cyan–orange, according to photoluminescence spectroscopy (Wongso et al. 2019).

RH was fully rinsed using DI water, dry, and crushed into powders from a 1.0 g sample (100 mesh). Before being dried in a vacuum at room temperature for 24 h, the powder was rinsed 3 times in 0.10 M HCl and DI water. A 100 mg dried RH grains sample was mixed with 20 mL DI water in a Teflon®-walled autoclave. The hydrothermal reaction was performed at 150 °C for 5 h. The hydrothermal reaction was performed at 150 °C. The mixture was filtered after the reaction. The supernatant, consisting RH-GQDs, was obtained by centrifuging the filtrate at 28,000 g force for 15 min. The synthesized GQDs were highly sensitive to Fe^3+^ ions, suggesting that they could be used for Fe^3+^ sensing (Wang et al. 2018a, b).

Arivuselvi et al. used a hydrothermal technique to make the carbon quantum dot from rice husk in 4 h at 200 °C. A 5.0 g sample of rice husks was completely rinsed in deionized (DI) water before being ground to powder (100 mesh). The powder was then processed for 2 h in a tube furnace at 600 °C in an N_2_ environment. Then, 0.89 g of RH ash (RHA), which contains carbon and silica, was taken. The RHA was treated with excess KOH and a magnetic stirrer for 10 min. RHA was transformed into a combination of RHC and potassium silicate throughout this procedure. The synthesized powder was then extensively rinsed with DI water and dried for 2 h at 80 °C. The resulting potassium silicate solution was collected and might be used to make various silicon-based functional materials. HNO_3_ was added slowly to a sample of 6 g of RHC mixed with 20 ml of H_2_SO_4_. The solution was allowed to settle in the bottom of the flask before being carefully rinsed in DI water and filtered through Whatman filter paper. The solution was maintained at 200° C for 4 h in a Teflon-lined autoclave. The resulting solution was then allowed to cool to ambient temperature before being filtered through Whatman filter paper. Finally, anneal for 2 h at 80 °C to remove any remaining water. RH-GQDs were finally ready to be powered (Arivuselvi and Ramalingam 2017).

The synthesis of RH-derived CQDs was performed, demonstrating how functional groups influenced the RH-derived CQDs. Husks were created so the hydrothermal carbonizing RH could go more smoothly. CQDs were dialyzed for one day with deionized water using visking tubes made of cellulose 10 M to improve the level of purity achieved. The following techniques were utilized to determine CQDs’ characteristics: FTIR, thermal analysis, XPS analysis, XRD, UV–Vis, and HRTEM (Abidin et al. 2020a, b).

## Conclusions and future perspectives

These materials can be made in a straightforward and cost-effective method. Several processes have been looked into to produce CNMs; however, greener ones with less waste material are gaining popularity. As a result, researchers are increasingly focused on building the latest techniques for manufacturing CNMs using simple, eco-friendly, cheap, and sustainable routes that use natural and abundant renewable resources, particularly agricultural waste. This chapter focuses on rice husk, an agricultural waste that can be used as a starting material to synthesize CNMs like graphene, CNTs, and CDs.

RH is a significant by-product of paddy rice milling and is produced in large quantities. The rice milling industry is facing a major challenge. When rice husk is used as a feedstock for the production of CNMs, it must be pretreated, which changes the reaction conditions and requirements. This limits the amount of rice husk that can be used to this restriction; we must create studies that do not need pre-treatment and make their application as straightforward as possible. Water pollution remediation, energy storage, catalysis, biomedical sensors, medication delivery, and bioimaging are just a few of the possibilities for rice husk CNMs.

Although several research projects on the use of RH and the result of its thermal breakdown, RHA, are currently in progress, most of these investigations have been carried out on a laboratory scale. RH/RHA is an excellent precursor for producing high-value-added CNMs that are used in practical applications since it originates from a natural, sustainable, and renewable source. RH/RHA is utilized extensively because it makes it possible to convert easily accessible agro-waste into things with additional value while lowering the amount of pollution produced.

We undertake and agree that the manuscript submitted to your journal has not been published elsewhere and has not been simultaneously submitted to other journals.

## Data Availability

Not applicable.

## Abbreviations

RH: Rice husk
CNMs: Carbon-based nanomaterials
AWM: Agricultural Waste Management
CNTs: Carbon nanotubes
RHA: Rice husk ash
SEM: Scanning electron microscope
TEM: Transmission electron microscopy
MWCNTs: Multiwalled carbon nanotubes
C-dots: Carbon dots
GQDs: Graphene quantum dots
PPy: Polypyrrole
SSA: Specific surface area
GO: Graphene oxide
PP: Polypropylene
PA 6: Polyamine-6
MA: Maleic anhydride
GRH: Husk-derived graphenes
GnP: Graphene nanoplatelets
RH-CG: Rice husk-derived corrugated graphene
CQDs: Carbon quantum dots
DFT: Density-functional theory
VOCs: Volatile organic compounds
HR-TEM: High-resolution transmission electron microscopy
EDX: Energy dispersive X-ray
FTIR: Fourier transform infrared
XPS: X-ray photo electron spectroscopy
XRD: X-ray diffraction analysis

## References

[CR1] Abdel Ghafar HH, Ali GAM, Fouad OA, Makhlouf SA (2015). Enhancement of adsorption efficiency of methylene blue on Co_3_O_4_/SiO_2_ nanocomposite. Desalination Water Treat.

[CR2] Abdelbary S, Abdelfattah H (2020). Modern trends in uses of different wastes to produce nanoparticles and their environmental applications.

[CR3] Abidin NH, Wongso V, Hui KC, Cho K, Sambudi NS, Ang WL, Saad B (2020). The effect of functionalization on rice-husks derived carbon quantum dots properties and cadmium removal. Water Process Eng.

[CR4] Abidin NH, Wongso V, Hui KC, Cho K, Sambudi NS, Ang WL, Saad B (2020). The effect of functionalization on rice-husks derived carbon quantum dots properties and cadmium removal. J Water Process Eng.

[CR5] Agamuthu P (2009) Challenges and opportunities in agro-waste management: an Asian perspective. In Inaugural meeting of first regional 3R forum in Asia, Nov 11 (pp. 11–12). University of Malasiya.

[CR6] Akhtar N, Emran MY, Shenashen MA,. Khalifa H, Osaka T, Faheem A, Homma T, Kawarada H, El-Safty SA (2017). Fabrication of photo-electrochemical biosensors for ultrasensitive screening of mono-bioactive molecules: the effect of geometrical structures and crystal surfaces. J Mater Chem B. 57985–7996. 10.1039/C7TB01803G. 10.1039/c7tb01803g 32264199

[CR7] Ali GAM, Fouad OA, Makhlouf SA (2013). Structural, optical and electrical properties of sol-gel prepared mesoporous Co_3_O_4_/SiO_2_ nanocomposites. J Alloy Compd.

[CR8] Ali GA, Manaf SA, Kumar A, Chong KF, Hegde G (2014). High performance supercapacitor using catalysis free porous carbon nanoparticles. J Phys D Appl Phys.

[CR9] Ali GAM, Tan LL, Jose R, Yusoff MM, Chong KF (2014). Electrochemical performance studies of MnO_2_ nanoflowers recovered from spent battery. Mater Res Bull.

[CR10] Ali GAM, Fouad OA, Makhlouf SA (2016). Electrical properties of cobalt oxide/silica nanocomposites obtained by sol-gel technique. Am J Eng Appl Sci.

[CR11] Ali GA, Divyashree A, Supriya S, Chong KF, Ethiraj AS, Reddy MV, Algarni H, Hegde G (2017). Carbon nanospheres derived from Lablab purpureus for high performance supercapacitor electrodes: a green approach. Dalton Trans.

[CR12] Ali GAM, Lih Teo EY, Aboelazm EAA, Sadegh H, Memar AOH, Shahryari-Ghoshekandi R, Chong KF (2017). Capacitive performance of cysteamine functionalized carbon nanotubes. Mater Chem Phys.

[CR13] Ali GAM, Habeeb OA, Algarni H, Chong KF (2018). CaO impregnated highly porous honeycomb activated carbon from agriculture waste: symmetrical supercapacitor study. J Mater Sci.

[CR14] Ali GAM, Barhoum A, Gupta VK, Nada AA, El-Maghrabi H, Kanthasamy R, Shaaban ER, Algarni H, Chong KF (2020). High surface area mesoporous silica for hydrogen sulphide effective removal. Curr Nano Sci.

[CR15] Allen MJ, Tung VC, Kaner RB (2010). Honeycomb carbon: a review of graphene. Chem Rev.

[CR16] Almeida SR, Elicker C, Vieira BM, Cabral TH, Silva AF, Sanches Filho PJ, Raubach CW, Hartwig CA, Mesko MF, Moreira ML, Cava S (2019). Black SiO_2_ nanoparticles obtained by pyrolysis of rice husk. Dyes Pig.

[CR17] An D, Guo Y, Zhu Y, Wang Z (2010). A green route to preparation of silica powders with rice husk ash and waste gas. Chem Eng J.

[CR18] Anantram M, Leonard F (2006). Physics of carbon nanotube electronic devices. Rep Prog Phys.

[CR19] Andas J, Adam F (2016). One-pot synthesis of nanoscale silver supported biomass-derived silica. Mater Today: Proceedings.

[CR20] Arivuselvi E, Ramalingam G (2017). Insight into the Bio-Approach of Claims for Quantum Dots by Using Bio-Waste (rice Husk).

[CR21] Asnawi M, Azhari S, Hamidon MN, Ismail I, Helina I (2018). Synthesis of carbon nanomaterials from rice husk via microwave oven. J Nanomater.

[CR22] Azizovna SM, Ivanovich CD, Zulkhair M (2017). Development of a method of obtaining graphene layers from rice husk. Func Nanostr Proc.

[CR23] Baker SN, Baker GA (2010). Luminescent carbon nanodots: emergent nanolights. Angew. Chem. Int Ed.

[CR24] Barhoum A, Shalan AE, El-Hout SI, Ali GAM, Abdelbasir SM, Abu Serea ES, Ibrahim AH, Pal K, Barhoum A, Bechelany M, Makhlouf A (2019). A broad family of carbon nanomaterials: classification, properties, synthesis, and emerging applications. Handbook of nanofibers.

[CR25] Bathla A, Narula C, Chauhan RP (2018). Hydrothermal synthesis and characterization of silica nanowires using rice husk ash: an agricultural waste. J Mater Sci Mater El.

[CR26] Baughman RH, Zakhidov AA, de Heer WA (2002). Carbon nanotubes–the route toward applications. Science.

[CR27] Bernd MGS (2017). Synthesis of carbon nanostructures by the pyrolysis of wood sawdust in a tubular reactor. J Market Res.

[CR28] Carbon filaments and nanotubes: common origins, differing, applications?, ed. Birὀ LP, Bernardo CA, Tibbetts GG, Larnbin PH, Kluwer Academic, Dordrecht, Netherlands, 2001.

[CR29] Biswas B, Pandey N, Bisht Y, Singh R, Kumar J, Bhaskar T (2017). Pyrolysis of agricultural biomass residues: comparative study of corn cob, wheat straw, rice straw and rice husk. Bioresour Technol.

[CR30] Chakroborty S, Nath N, Soren S, Barik A, Kaur K (2023). Plasmonic-based TiO_2_ and TiO_2_ nanoparticles for photocatalytic CO_2_ to methanol conversion in energy applications: current status and future prospects. Top. Catal. 1-1410.1007/s11244-023-01816-5

[CR31] Champagne ET, Wood DF, Juliano BO, Bechtel DB (2004). The rice grain and its gross composition. Rice Chem Technol.

[CR32] Chaudhary DS, Jollands MC (2004). Characterization of rice hull ash. J Appl Polym Sci.

[CR33] Che Othman FE, Ismail MS, Yusof N, Samitsu S, Yusop MZ, Tajul Arifin NF, Alias NH, Jaafar J, Aziz F, Wan Salleh WN, Ismail AF (2020). Methane adsorption by porous graphene derived from rice husk ashes under various stabilization temperatures. Carbon Lett.

[CR34] Chen F (2016). Facile synthesis of few-layer graphene from biomass waste and its application in lithium ion batteries. J Electroanal Chem.

[CR35] Choi W, Lahiri I, Seelaboyina R, Kang YS (2010). Synthesis of graphene and its applications: a review. Crit Rev Solid State Mater Sci.

[CR36] Chu H, Wei L, Cui R, Wang J, Li Y (2010). Carbon nanotubes combined with inorganic nanomaterials: preparations and applications. Coord Chem Rev.

[CR37] Dai H (2002). Carbon nanotubes: synthesis, integration, and properties. Acc Chem Res.

[CR38] de Volder MFL, Tawfick SH, Baughman RH, Hart AJ (2013). Carbon nanotubes: present and future commercial applications. Science.

[CR39] Emran MY, Khalifa H, Gomaa H, Shenashen MA, Akhtar N, Mekawy M, Faheem A, El-Safty SA (2017). Hierarchical CN doped NiO with dual-head echinop flowers for ultrasensitive monitoring of epinephrine in human blood serum. Microchim Acta.

[CR40] Emran MY, Mekawy M, Akhtar N, Shenashen MA, El-Sewify IM, Faheem A, El-Safty SA (2018). Broccoli-shaped biosensor hierarchy for electrochemical screening of noradrenaline in living cells. Biosens Bioelectron.

[CR41] Emran MY, Shenashen MA, Abdelwahab AA, Abdelmottaleb M, El-Safty SA (2018). Facile synthesis of microporous sulfur-doped carbon spheres as electrodes for ultrasensitive detection of ascorbic acid in food and pharmaceutical products. New J Chem.

[CR42] Emran MY, Shenashen MA, Abdelwahab AA, Abdelmottaleb M, Khairy M, El-Safty SA (2018). Nanohexagonal Fe _2_ O _3_ electrode for one-step selective monitoring of dopamine and uric acid in biological samples. Electrocatalysis.

[CR43] Emran MY, Shenashen MA, Abdelwahab AA, Khalifa H, Mekawy M, Akhtar N, Abdelmottaleb M, El-Safty SA (2018). Design of hierarchical electrocatalytic mediator for one step, selective screening of biomolecules in biological fluid samples. J Appl Electrochem.

[CR44] Emran MY, Shenashen MA, Mekawy M, Azzam AM, Akhtar N, Gomaa H, Selim MM, Faheem A, El-Safty SA (2018). Ultrasensitive in-vitro monitoring of monoamineneurotransmitters from dopaminergic cells. Sens Actuators b: Chem.

[CR45] Emran MY, Shenashen MA, Morita H, El-Safty SA (2018). 3D-ridge stocked layers of nitrogen-doped mesoporous carbon nanosheets for ultrasensitive monitoring of dopamine released from PC12 cells under K + stimulation. Adv Healthc Mater.

[CR46] Emran MY, Shenashen MA, Morita H, El-Safty SA (2018). One-step selective screening of bioactive molecules in living cells using sulfur-doped microporous carbon. Biosens Bioelectron.

[CR47] Emran MY, El-Safty SA, Shenashen MA, Minowa T (2019). A well-thought-out sensory protocol for screening of oxygen reactive species released from cancer cells. Sens Actuators, B Chem.

[CR48] Fadllan A, Marwoto P, Aji MP, Susanto S, Iswari RS (2017). Synthesis of carbon nanodots from waste paper with hydrothermal method. AIP Conf Proc.

[CR49] Fang M, Yang L, Chen G, Shi Z, Luo Z, Cen K (2004). Experimental study on rice husk combustion in a circulating fluidized bed. Fuel Process Technol.

[CR50] Fathy NA (2017). Carbon nanotubes synthesis using carbonization of pretreated rice straw through chemical vapor deposition of camphor. RSC Adv.

[CR51] Fouad OA, Makhlouf SA, Ali GAM, El-Sayed AY (2011). Cobalt/silica nanocomposite via thermal calcination-reduction of gel precursors. Mater Chem Phys.

[CR52] Fouad OA, Ali GAM, El-Erian MAI, Makhlouf SA (2012). Humidity sensing properties of cobalt oxide/silica nanocomposites prepared via sol-gel and related routes. NANO.

[CR53] Ghaly A, Mansaray K (1999). Comparative study on the thermal degradation of rice husks in various atmospheres. Energy Sources.

[CR54] Gomaa H, Khalifa H, Selim M, Shenashen M, Kawada S, Alamoudi AS, Azzam A, Alhamid A, El-Safty S (2017). Selective, photoenhanced trapping/detrapping of arsenate anions using mesoporous blobfish head TiO_2_ monoliths. ACS Sustain Chem Eng.

[CR55] Gomaa H, Shenashen M, Yamaguchi H, Alamoudi A, El-Safty S (2018). Extraction and recovery of Co ^2 +^ ions from spent lithium-ion batteries using hierarchical mesosponge-Al _2_O _3_ monolith extractors. Green Chem.

[CR56] Greil P (2015). Perspectives of nanocarbon based engineering materials. Adv Eng Mater.

[CR57] Griffin S, Masood MI, Nasim MJ, Sarfraz M, Ebokaiwe AP, Schäfer KH, Keck CM, Jacob C (2018) Natural nanoparticles: a particular matter inspired by nature. Antioxidants 73. 10.3390/antiox7010003. 10.3390/antiox7010003 PMC5789313 29286304

[CR58] Global Rice Science Partnership. Rice Agri-Food System CRP, RICE (International Rice Research Institute, 2016.

[CR59] Guan L, Pan L, Peng T, Gao C, Zhao W, Yang Z, Hu H, Wu M (2019). Synthesis of biomass-derived nitrogen-doped porous carbon nanosheests for high-performance supercapacitors, ACS Sustain. Chem Eng.

[CR60] Hadiya UB, Javed F, Jadeja KY (2018). Approach towards enhancing sanitation in godavadi village through sustainable solid waste management. Int Res J Eng Technol.

[CR61] Hai HT, Tuyet NT (2010) Benefits of the 3R approach for Agricultural Waste Management (AWM) in Vietnam: under the framework of joint project on Asia Resource Circulation Research. Institute for Global Environmental Strategies.

[CR62] Harrison BS, Atala A (2007). Carbon nanotube applications for tissue engineering. Biomaterials.

[CR63] Hegde G, Abdul Manaf SA, Kumar A, Ali GA, Chong KF, Ngaini Z, Sharma KV (2015). Biowaste sago bark based catalyst free carbon nanospheres: waste to wealth approach. ACS Sustain Chem Eng.

[CR64] Homaeigohar S, Elbahri M (2017). Graphene membranes for water desalination. NPG Asia Materials.

[CR65] Huston M, DeBella M, DiBella M, Gupta A (2021). Green Synthesis of Nanomaterials. Nanomater Nanomater.

[CR66] Iijima S (1991). Helical microtubules of graphitic carbon. Nature.

[CR67] Ismail MS, Yusof N, Yusop MZM, Ismail AF, Jaafar J, Aziz F, Karim Z A (2019) Synthesis and characterization of graphene derived from rice husks. Malaysian J.Fundam. Appl. Sci. 15 516–521. https://www.researchgate.net/publication/336826132.

[CR68] Jha A, Ghorai UK, Banerjee D, Mukherjee S, Chattopadhyay KK (2013). Surface modification of amorphous carbon nanotubes with copper phthalocyanine leading to enhanced field emission. RSC Adv..

[CR69] Jiang JW, Leng J, Li J, Guo Z, Chang T, Guo X, Zhang T (2017). Twin graphene: a novel two-dimensional semiconducting carbon allotrope. Carbon.

[CR70] Jiang XH (2010) The research on application of the rice husk ash. Master Dissertation, Harbin Institute of Technology, People’s Republic of China.

[CR71] Jin H, Wu S, Li T, Bai Y, Wang X, Zhang H, Xu H, Kong C, Wang H (2019). Synthesis of porous carbon nano-onions derived from rice husk for high-performance supercapacitors. Appl Surf Sci.

[CR72] Jing S, Zhao Y, Sun RC, Zhong L, Peng X (2019). Facile and high-yield synthesis of carbon quantum dots from biomass-derived carbons at mild condition. ACS Sustain Chem Eng.

[CR73] Jung SH, Kang BS, Kim JS (2008). Production of bio-oil from rice straw and bamboo sawdust under various reaction conditions in a fast pyrolysis plant equipped with a fluidized bed and a char separation system. J Anal Appl Pyr.

[CR74] Kalita E, Nath BK, Deb P, Agan F, Islam MR, Saikia K (2015). High quality fluorescent cellulose nanofibers from endemic rice husk: isolation and characterization. Carbohydr Polym.

[CR75] Kim MJ, Yoon SH, Choi E, Gil B (2008). Comparison of the adsorbent performance between rice hull ash and rice hull silica gel according to their structural differences. LWT Food Sci Technol.

[CR76] Kokorina AA, Prikhozhdenko ES, Sukhorukov GB, Sapelkin AV, Goryacheva IY (2017). Luminescent carbon nanoparticles: synthesis, methods of investigation, applications. Russ Chem Rev.

[CR77] Kumar M, Sachdeva A, Garg RK, Singh S (2020). Synthesis and characterization of graphene prepared from rice husk by a simple microwave process. InNano Hybrids and Composites. Trans Tech Publ Ltd.

[CR78] Kure N, Hamidon MN, Azhari S, Mamat N, Yusoff H, Isa B, Yunusa Z (2017) Simple microwave-assisted synthesis of carbon nanotubes using polyethylene as carbon precursor. J. Nanomat10.1155/2017/2474267

[CR79] Leung AC, Lam E, Chong J, Hrapovic S, Luong JH (2013). Reinforced plastics andaerogels by nanocrystalline cellulose. J Nanopart Res.

[CR80] Lim SY, Shen W, Gao Z (2015). Carbon quantum dots and their applications. Chem Soc Rev.

[CR81] Liou TH, Yang CC (2011). Synthesis and surface characteristics of nanosilica produced from alkali-extracted rice husk ash. Mater Sci Eng B.

[CR82] Liu N, Huo K, McDowell MT, Zhao J, Cui Y (2013). Rice husks as a sustainable source of nanostructured silicon for high performance Li-ion battery anodes. Sci Rep.

[CR83] Mahmoud ME, Fekry NA, Abdelfattah AM (2020). A novel nanobiosorbent of functionalized graphene quantum dots from rice husk with barium hydroxide for microwave enhanced removal of lead (II) and lanthanum (III). Bioresource Technol.

[CR84] Manpetch P, Singhapong W, Jaroenworaluck A (2022). Synthesis and characterization of a novel composite of rice husk-derived graphene oxide with titania microspheres (GO-RH/TiO2) for effective treatment of cationic dye methylene blue in aqueous solutions. Environ Sci Pollut Res.

[CR85] Mathur L, Hossain SS, Majhi MR, Roy PK (2018). Synthesis of nano-crystalline forsterite (Mg_2_SiO_4_) powder from biomass rice husk silica by solid-state route. Bol Soc Esp Ceram.

[CR86] Matsumoto K, Kamiya K, Ito S, Kawabata S, Inada M (2018). Luminescent Si nanomaterials prepared from rice husks of agricultural waste. Trans Mater Res Soc Japan.

[CR87] Mohamed R, Mkhalid I, Barakat M (2015). Rice husk ash as a renewable source for the production of zeolite NaY and its characterization. Arab J Chem.

[CR88] Moon RJ, Martini A, Nairn J, Simonsen J, Youngblood J (2011). Cellulose nanomaterials review: structure, properties and nanocomposites. Chem Soc Rev.

[CR89] Mor S, Manchanda CK, Kansal SK, Ravindra K (2017). Nanosilica extraction from processed agricultural residue using green technology. J Clean Prod.

[CR90] Moraes CA, Fernandes IJ, Calheiro D, Kieling AG, Brehm FA, Rigon MR, Berwanger Filho JA, Schneider IA, Osorio E (2014). Review of the rice production cycle: by-products and the main applications focusing on rice husk combustion and ash recycling. Waste Manag Res.

[CR91] Mukherjee A, Majumdar S, Servin AD, Pagano L, Dhankher OP, White JC (2016). Carbon nanomaterials in agriculture: a critical review. Front Plant Sci.

[CR92] Muramatsu H, Kim YA, Yang KS, Cruz-Silva R, Toda L, Yamada T, Terrones M, Endo M, Hayashi T, Saitoh H (2014). Ricehusk-derived graphene with nano-sized domains and clean edges. Small.

[CR93] Naddaf M, Kafa H, Ghanem I (2019). Extraction and characterization of nano-silica from olive stones. Silicon.

[CR94] Nath N, Kumar A, Chakroborty S, Soren S, Barik A, Pal K, de Souza Jr FG (2023). Carbon nanostructure embedded novel sensor implementation for detection of aromatic volatile organic compounds: an organized review. ACS Omega.

[CR95] Nath N, Chakroborty S, Pal K, Barik A, Mishra, NP, Kralj S (2023a). Recent advances in plasmonic enhanced nanocatalyst for oxidation of alcohol. Top. Catal. 1–11. 10.1007/s11244-023-01839-y

[CR96] Nath N, Chakroborty S, Vishwakarma DP, Goga G, Yadav AS, Mohan R (2023c) Recent advances in sustainable nature-based functional materials for biomedical sensor technologies. Environ. Sci. Pollut. Res. 1–25. 10.1007/s11356-023-26135-w 10.1007/s11356-023-26135-w PMC9975880 36857000

[CR97] Novoselov KS, Geim AK, Morozov SV, Jiang D (2004). Electric field effect in atomically thin carbon films. Science.

[CR98] Obi FO, Ugwuishiwu BO, Nwakaire JN (2016). Agricultural waste concept, generation, utilization and management. Niger J Technol.

[CR99] Omatola KM, Onojah AD (2009) Elemental analysis of rice husk ash using X-ray fluorescence technique. Int. J. Phys. Sci. 4 :189–193. http://www.academicjournals.org/IJPS.

[CR100] Ouyang D, Chen K (2003). SEM/TEM study on the microstructure of rice husk ash andnano-SiO_2_ in it. J Chin Electron Microsc Soc.

[CR101] Pal K, Chakroborty S, Nath N (2022). Limitations of nanomaterials insights in green chemistry sustainable route: review on novel applications. Green Process Synth.

[CR102] Pal K, Chakroborty S, Panda P, Nath N, Soren S (2022). Environmental remediation through wastewater management via hybrid nanocomposite matrix–a organized review. Environ Sci Pollut Res.

[CR103] Patil R, Dongre R, Meshram J (2014) Preparation of silica powder from rice husk, IOSR J. Appl. Chem. 27: 26–29. http://www.iosrjournals.org/.

[CR104] Pek YS, Wan AC, Shekaran A, Zhuo L, Ying JY (2008). A thixotropic nanocomposite gel for three-dimensional cell culture. Nat Nanotechnol.

[CR105] Pouroutzidou GK, Theodorou GS, Kontonasaki E, Tsamesidis I, Pantaleo A, Patsiaoura D, Papadopoulou L, Rhoades J, Likotrafiti E, Lioutas CB (2019). Effect of ethanol/TEOS ratio sand amount of ammonia on the properties of copper-doped calcium silicate nanoceramics. J Mater Sci Mater Med.

[CR106] Praneetha S, Murugan AV (2015). Development of sustainable rapid microwave assisted process for extracting nanoporous Si from earth abundant agricultural residues and their carbon-based nanohybrids for Lithium energy storage. ACS Sustain Chem Eng.

[CR107] Prasek J, Drbohlavova J, Chomoucka J, Hubalek J, Jasek O, Adam V, Kizek R (2011). Methods for carbon nanotubes synthesis—review. J Mater Chem.

[CR108] Purkait T (2017). Large area few-layer graphene with scalable preparation from waste biomass for high-performance supercapacitor. Sci Rep.

[CR109] Purwaningsih H, Raharjo S, Pratiwi VM, Susanti D, Purniawan A (2019). Porous silica nanomaterial derived from organic waste Rice husk as highly potential drug delivery material. Mater Sci Forum.

[CR110] Qi HJ, Teo KBK, Lau KKS, Boyce MC, Milne WI, Robertson J, Gleason KK (2003). Determination of mechanical properties of carbon nanotubes and vertically aligned carbon nanotube forests using nanoidentation. J Mech Phys Solids.

[CR111] Quispe I, Navia R, Kahhat R (2017). Energy potential from rice husk through direct combustion and fast pyrolysis: a review. Waste Manag.

[CR112] Rhee I, Kim YA, Shin GO, Kim JH, Muramatsu H (2015). Compressive strength sensitivity of cement mortar using rice husk-derived graphene with a high specific surface area. Constr Build Mater.

[CR113] Rhee I, Lee JS, Kim YA, Kim JH, Kim JH (2016). Electrically conductive cement mortar: incorporating rice husk-derived high-surface-area graphene. Constr Build Mater.

[CR114] Rhee I, Lee JS, Kim JH, Kim YA (2017). Thermal performance, freeze-and-thaw resistance, and bond strength of cement mortar using rice husk-derived graphene. Constr Build Mater.

[CR115] Sahoo M, Vishwakarma S, Panigrahi C, Kumar J (2021). Nanotechnology: Current applications and future scope in food. Food Front.

[CR116] Sankar S, Lee H, Hyun J, Kim A, Abu Talha AA, Inamdar A, Kim H, Lee S, Im H, Kim DY (2017). New J Chem.

[CR117] Sekar S, Ahmed AT, Sim DH, Lee S (2022). Extraordinarily high hydrogen-evolution-reaction activity of corrugated graphene nanosheets derived from biomass rice husks. Int J Hydrogen Energy.

[CR118] Serp P, Figueiredo JL (2009) 7 Carbon materials for catalysis, ed. John Wiley & Sons Inc, New Jersey,

[CR119] Seyfferth AL, Morris AH, Gill R, Kearns KA, Mann JN, Paukett M, Leskanic C (2016). Soil incorporation of silica-rich rice husk decreases inorganic arsenic in rice grain. J Agric Food Chem.

[CR120] Sharon M, Mewada A (2018) Carbon dots: discovery, synthesis and characterization, 1–45. 10.1002/9781119460435.ch1

[CR121] Singh P, Bahadur J, Pal K (2017). One-step one chemical synthesis process of graphene from rice husk for energy storage applications. Graphene.

[CR122] Singh P, Srivastava S, Chakrabarti P, Singh SK (2017). Nanosilica based electrochemical biosensor: a novel approach for the detection of platelet-derived microparticles. Sens Actuators b: Chem.

[CR123] Siqueira JR, Oliveira ON (2017) Carbon-based nanomaterials. Nanostructures. A.L. Da Róz, M. Ferreira, F. de Lima Leite, Oliveira, O.N., Eds.; William Andrew Publishing: Norwich, UK, 233–249.

[CR124] Stroeven P, Dai Bui D, Sabuni E (1999). Ash of vegetable waste used for economic production of low to high strength hydraulic binders. Fuel.

[CR125] Sujiono EH (2020). Graphene oxide based coconut shell waste: synthesis by modified Hummers method and characterization. Heliyon.

[CR126] Tang L, Cheng J (2013). Nonporous silica nanoparticles for nanomedicine application. Nano Today.

[CR127] Tanniru M, Tambe P (2022). Selective localization of rice husk derived graphene in reactive compatibilized PP/PA6 blends: influence on morphology, interface and mechanical properties. Fuller Nanotub Carbon Nanostr.

[CR128] Tao Z (2014). Mesoporous silica-based nanodevices for biological applications. RSC Adv.

[CR129] Thalji MR, Ali GAM, Algarni H, Chong KF (2019). Al3+ ion intercalation pseudocapacitance study of W_18_O_49_ nanostructure. J Power Sour.

[CR130] Thongsai N, Tanawannapong N, Praneerad J, Kladsomboon S, Jaiyong P, Paoprasert P (2019). Real-time detection of alcohol vapors and volatile organic compounds via optical electronic nose using carbon dots prepared from rice husk and density functional theory calculation. Colloids Surf a: Physicochem Eng Asp.

[CR131] Ugheoke I.B, Mamat O (2012) A critical assessment and new research directions of rice husk silica processing methods and properties. Maejo. Int. J. Sci. Technol. 6: 430. http://www.mijst.mju.ac.th/.

[CR132] Umeda J, Kondoh K (2010). High-purification of amorphous silica originated from rice husks by combination of polysaccharide hydrolysis and metallic impurities removal. Ind Crops Prod.

[CR133] Vanlalveni C, Lallianrawna S, Biswas A, Selvaraj M, Changmai B, Rokhum SL (2021). Green synthesis of silver nanoparticles using plant extracts and their antimicrobial activities: a review of recent literature. RSC Adv.

[CR134] Vargas C, Simarro R, Reina JA, Bautista LF, Molina MC, Gonz’alez-Benítez N (2019). New approach for biological synthesis of reduced graphene oxide. Biochem Eng J.

[CR135] Villarreal CC, Pham T, Ramnani P, Mulchandani A (2017). Carbon allotropes as sensors for environmental monitoring. Curr Opin Electrochem.

[CR136] Vlaev L, Markovska I, Lyubchev L (2003). Non-isothermal kinetics of pyrolysis of rice husk. Thermo Chim Acta.

[CR137] Wang Z, Ogata H, Morimoto S, Ortiz-Medina J, Fujishige M, Takeuchi K, Muramatsu H, Hayashi T, Terrones M, Hashimoto Y (2015). Nanocarbons from rice husk by microwave plasma irradiation: from graphene and carbon nanotubes to graphenated carbon nanotube hybrids. Carbon.

[CR138] Wang Z, Ogata H, Morimoto S, Ortiz-Medina J, Fujishige M, Takeuchi K, Muramatsu H, Hayashi T, Terrones M, Hashimoto Y, Endo M (2015). Nanocarbons from rice husk by microwave plasma irradiation: from graphene and carbon nanotubes to graphenated carbon nanotube hybrids. Carbon.

[CR139] Wang Z, Yu J, Zhang X, Li N, Liu B, Li Y, Wang Y, Wang W, Li Y, Zhang L (2016). Large-scale and controllable synthesis of graphene quantum dots from rice husk biomass: a comprehensive utilization strategy. ACS Appl Mater Interfaces.

[CR140] Wang Z, Liu J, Wang W, Wei Z, Wang F, Gong P, Wang J, Li N, Liu B, Zhang Z, Wang W (2017). Photoluminescent carbon quantum dot grafted silica nanoparticles directly synthesized from rice husk biomass. Mater Chem B.

[CR141] Wang W, Wang Z, Liu J, Peng Y, Yu X, Wang W, Zhang Z, Sun L (2018). One-pot facile synthesis of graphene quantum dots from rice husks for Fe^3+^ sensing. Ind Eng Chem Res.

[CR142] Wang Z, Shen D, Wu C, Gu S (2018). State-of-the-art on the production and application of carbon nanomaterials from biomass. Green Chem.

[CR143] Wongso V, Sambudi NS, Sufian S, Abdullah B (2019). The effect of pH in the synthesis of carbon quantum dots from rice husk on their photoluminescence properties. IOP Conf Ser: Earth Environ Sci.

[CR144] Xu WT, Lo TY, Memon SA (2012). Microstructure and reactivity of rich husk ash. Constr Build Mater.

[CR145] Yu D, Nagelli E, Du F, Dai L (2010). Metal-free carbon nanomaterials become more active than metal catalysts and last longer. J Phys Chem Lett.

[CR146] Zhang M, Li J (2009). Carbon nanotube in different shapes. Mater Today.

[CR147] Zhang Z, Gonzalez AM, Davies EG, Liu Y (2012). Agricultural wastes. Water Environ Res.

[CR148] Zhang H, Zhang X, Sun X, Ma Y (2013). Shape-controlled synthesis of nanocarbons through direct conversion of carbon dioxide. Sci Rep.

[CR149] Zhang Y-C, You Y, Xin S, Yin Y-X, Zhang J, Wang P, Zheng X, Cao F-F, Guo Y-G (2016). Rice husk-derived hierarchical silicon/nitrogen-doped carbon/carbon nanotube spheres as low-cost and high-capacity anodes for lithium-ion batteries. Nano Energy.

[CR150] Zhu J (2012). Synthesis of multiwalled carbon nanotubes from bamboo charcoal and the roles of minerals on their growth. Biomass Bioenerg.

[CR151] Zhu L, Shen D, Wu C, Gu S (2020). State-of-the-art on the preparation, modification, and application of biomass-derived carbon quantum dots. Ind Eng Chem Res.

[CR152] Zong S, Zhang Y, Xaba MS, Liu X, Chen A (2020). N-doped porous carbon nanotubes derived from polypyrrole for supercapacitors with high performance. J Anal Appl Pyrolysis.

[CR153] Zou Y, Yang T (2019) Rice husk, rice husk ash and their applications, in rice bran and rice bran oil chemistry, processing and utilization, 207–246. 10.1016/B978-0-12-812828-2.00009-3.

